# 2008 Nobel prize in Medicine for discoverers of HIV

**DOI:** 10.1186/1742-4690-5-91

**Published:** 2008-10-14

**Authors:** Andrew ML Lever, Ben Berkhout

**Affiliations:** 1 University of Cambridge, Department of Medicine, Addenbrooke's Hospital, UK; 2 University of Amsterdam, Laboratory of Experimental Virology, Academic Medical Center, The Netherlands

## Abstract

Françoise Barré-Sinoussi and Luc Montagnier, codiscoverers of HIV, the causative agent of AIDS, have been awarded the 2008 Nobel Prize in Physiology or Medicine. They share this prize with Harald zur Hausen who was responsible for establishing the link between human papilloma virus infection and cervical carcinoma.

## Editorial

Nobel prizes have often attracted controversy and controversial figures. William Ramsay was awarded the Nobel prize in Chemistry for the discovery of inert gases and subsequently went on to claim chemical transmutation of elements. Individual, institutional and national pride are all at stake when they are announced. One small Cambridge college boasts unashamedly of having been the workplace of no less than four Nobel Laureates including Perutz, Kendrew and Klug. Their value is more than financial and the method of their choosing somewhat mysterious and, to some peoples' minds flawed. Arrhenius having won in 1903, reputedly then manipulated future choices in favour of his friends and attempted (unsuccessfully) to exclude some very significant scientific figures such as Paul Ehrlich.

This year virology has been honoured and three scientists chosen who worked with two very different viruses. Harald zur Hausen, professor emeritus of the German Cancer Research Center in Heidelberg (Germany) identified the link between human papilloma virus (HPV) infection and cervical cancer. It is timely that with the recent development of a safe and effective vaccine he should be recognised for first identification of this human pathogenic virus. He made several particularly noteworthy findings in papilloma virus research. The earliest was the recognition that there are multiple HPV genotypes, particularly that HPVs that cause non-genital warts are distinct from those that cause genital warts. His most important finding was the identification and molecular cloning of the HPV types 16 and 18 genomes, and that a majority of cervical cancers contained DNA from these two HPV types. A third critical finding was that the HPV DNA becomes integrated into the host genome in cervical cancer cell lines and that the viral E6 and E7 (onco)genes are preferentially retained and expressed in the tumors. His work is even more noteworthy because it was carried out in the 1980's when there was considerable scepticism whether viruses in general, and HPV in particular, caused any common cancers. Professor zur Hausen has continued to run a highly productive laboratory since these seminal early findings. No controversy thus far.

Retroviruses have generated more than their share of Nobel prizes. Peyton Rous for retroviral oncogenesis (1966), Temin and Baltimore for reverse transcriptase (1975) and Varmus and Bishop again for oncogenes that were originally described for retroviruses (1989). The human immunodeficiency virus (HIV) as the largest public health problem in the world was destined to follow sooner or later. The difference is that, unlike in papilloma viruses, and whilst antiviral treatment has made astonishing progress in 20 years, we are no nearer a cure and certainly years from a vaccine (should one ever be possible) against HIV. The Nobel committee have chosen to honour the scientists who most people agree were the first to actually isolate the virus at the Pasteur Institute in Paris (France) in 1983: Françoise Barré-Sinoussi and Luc Montagnier. Following medical reports of a novel immunodeficiency syndrome in 1981, the search for a causative agent was on. The French researchers isolated and cultured cells from lymph nodes of patients with the lymphadenopathy characteristic of the early stage of acquired immune deficiency. They detected activity of the retroviral enzyme reverse transcriptase, a direct sign of retrovirus replication. They also observed retroviral particles budding from the infected cells. Isolated virus infected and killed lymphocytes from healthy donors and reacted with antibodies from infected patients. By 1984, Barré-Sinoussi and Montagnier had obtained several isolates of the novel human retrovirus from sexually infected individuals, haemophiliacs, mother to infant transmissions and transfused patients.

Nobel prizes can be split between a maximum of three individuals and sometimes a deserving fourth person has been omitted on what appear to be relatively tough pragmatic grounds. Had the Nobel committee decided this year that the prize was for the discovery of HIV alone there would have been only one possible third candidate. That they didn't may be perceived in some quarters as a more pointed statement. However the subjects into which the prize is divided may be very diverse. Peyton Rous shared his with Charles Huggins, honoured for his contribution to hormonal treatment of cancer. Luc Montagnier (perhaps with a winner's magnanimity) has said that the obvious third recipient should have been Robert Gallo. Others will quote Gallo's identification of HTLV-1 with Bernie Poiesz and the fact that many of the techniques used to grow viruses like HIV were dependent on discoveries from the Gallo lab as were some of the earliest blood tests for the virus. Had the Nobel committee honoured only those who contributed to the discovery of HIV rather than HPV as well the picture may have been different. Instead they chose to reward the two scientists most responsible for the first isolation of the virus. These two indeed deserve our congratulations. It would be churlish however not to acknowledge that the work of others, notably the Gallo lab – but also other scientists working at the time, and since – made very significant contributions without which it is likely that the unprecedented advances we have seen in our understanding and successful therapy of HIV might have been much slower in coming.

## Competing interests

The authors declare that they have no competing interests.

## Authors' contributions

Both authors contributed equally to the work.

